# AmpliSeq Transcriptome of Laser Captured Neurons from Alzheimer Brain: Comparison of Single Cell Versus Neuron Pools

**DOI:** 10.14336/AD.2019.0225

**Published:** 2019-12-01

**Authors:** Wenjun Deng, Changhong Xing, Rob David, Diego Mastroeni, MingMing Ning, Eng H Lo, Paul D Coleman

**Affiliations:** ^1^Neuroprotection Research Laboratories, Departments of Radiology and Neurology, Massachusetts General Hospital, Harvard Medical School, Charlestown, MA 02192, USA; ^2^Clinical Proteomics Research Center, Department of Neurology, Massachusetts General Hospital, Harvard Medical School, Boston, MA 02114, USA; ^3^Department of Pathology, University of Texas Southwestern Medical Center, Dallas, TX 75390, USA; ^4^Thermo-Fisher Scientific, Salem, MA 02114, USA; ^5^ASU-Banner Neurodegenerative Disease Research Center, Biodesign Institute, Arizona State University, Tempe, AZ 85281, USA

**Keywords:** AmpliSeq transcriptome, single neuron, Alzheimer's disease

## Abstract

Alzheimer’s disease (AD) is the most common cause of dementia in older adults. However, the pathogenesis of AD remains to be fully understood and clinically effective treatments are lacking. Recent advances in single cell RNA sequencing offers an opportunity to characterize the heterogeneity of cell response and explore the molecular mechanism of complex diseases at a single cell level. Here, we present the application of the Ion AmpliSeq transcriptome approach to profile gene expression in single laser captured neurons as well as pooled 10 and 100 neurons from hippocampal CA1 of AD brains versus matching normal aged brains. Our results demonstrated the high sensitivity and high genome coverage of the AmpliSeq transcriptome in single cell sequencing. In addition to capturing the known changes related to AD, our data confirmed the diversity of neuronal profiles in AD brain, which allow the potential identification of single cell response that might be hidden in population analyses. Notably, we also revealed the extensive inhibition of olfactory signaling and confirmed the reduction of neurotransmitter receptors in AD hippocampus. We conclude that although single neuron data show more variance than data from 10 or 100 pooled neurons, single neuron data can be informative. These findings support the utility of the Ion AmpliSeq method for obtaining and analyzing gene expression data from single defined laser captured neurons.

Alzheimer’s disease (AD) is a degenerative brain disease characterized by progressive cognitive impairment and eventually loss of independence. Over the past several decades, intensive research has revealed unique markers for AD diagnosis and progression monitoring, which include the formation of beta-amyloid (Aβ) plaques and neurofibrillary tangles, Tau hyperphosphorylation and apolipoprotein E (APOE) genotypes [1-5]. However, since the brain’s cognitive function involves the coordinated action of diverse sets of differentiated cell populations [6], the presentation and progression of AD demonstrates substantial variations in cognitive profile, onset age, and decline rate, and the molecular mechanisms leading to cognitive decline in neurodegeneration remain to be fully defined [7-11]. Therefore, understanding the differential gene expression among single neurons instead of profiling the average expression pattern from pooled samples in complex brain tissue may help to dissect the heterogeneous neural alterations in neurodegeneration and expand our knowledge on AD pathogenesis [12-14].

The possibility of obtaining expression profile from single neurons has been demonstrated since early 1990s [15, 16]. With the advances in RNA sequencing technology, it now becomes possible to precisely profile the genome of thousands of cells in a single assay. Recently, Thermo-Fisher Scientific developed an accurate and sensitive RNA sequencing technology, the Ion AmpliSeq transcriptome approach, which is capable of simultaneously amplifying and sequencing over 20,000 pre-defined genes with as little as 10 ng RNA input [17, 18]. With the advantage of targeted nature and small amplicon size (~150 bp), the turnaround time of Ion AmpliSeq technology is shorter than traditional whole transcriptome RNA sequencing and the number of raw reads assigned to each gene is smaller, which may facilitate experiment operation and data management, particularly when dealing with large dataset. However, up to now, the feasibility of AmpliSeq technology in single cell sequencing has never been evaluated, especially in terms of potentially resolving the heterogeneity of responses in the central nervous system. Here, we present the application of Ion AmpliSeq technology to the determination of expression profiles of single laser captured neurons in Alzheimer’s disease (AD) brain versus control neurons from normal aged brain. We focused on the CA1 region of the hippocampus because this is a brain area critical for learning and memory and one that demonstrates rapid neuron loss early on in AD progression. We also compared single neuron profiles to the gene expression obtained from the same tissue sections by concatenating 10 and 100 neurons per reaction. Our results confirmed the potential of the Ion AmpliSeq approach in profiling the whole transcriptome of single neuron and capturing cell-to-cell variation in gene expression. We also provided evidence that single cell sequencing can be more informative in potentially revealing heterogenous AD related neural alterations, which may be hidden when profiling average gene responses in pooled cell populations.

## MATERIALS AND METHODS

### Human subjects

Frozen unfixed tissue containing Samples of human CA1 hippocampus were secured from one AD (Braak IV) and one ND case (Braak III). Subjects were obtained at autopsy at the Banner Sun Health Research Institute Tissue Bank (BSHRI) in accordance with local IRB approval. BSHRI is a NIA AD Center Brain Bank that features extremely short postmortem intervals (PMIs) (mean of 2.8 hours in over 20 years of operation [19, 20], and very high-quality RNA (RIN of 8.5), as well as the detailed annotation required of NIA AD Centers. Cognitive status of all cases was evaluated antemortem by board-certified neurologists, and postmortem examination by a board-certified neuropathologist resulting in a consensus diagnosis using standard NIH AD Center criteria for AD or neurologically normal, non-demented elderly control. The AD and ND cases were well matched for age (AD: 71 years; ND: 71 years), gender (2 males), postmortem interval (PMI) (AD: 2.1 hours; ND: 2.2 hours) and ApoE genotype E3/E3. RIN values for AD subject was 7.7 and ND subject 7.9.

### Laser capture of pyramidal neurons

Frozen brain sections were cut at 15um, stained with 1% neutral red (Fisher Scientific) and mounted onto PEN slides required for laser capture microdissection. Immediately after staining sections were dipped in 100% ethanol and loaded onto a Leica AS-LMD laser capture microscope. Pyramidal neurons were identified by their characteristic size, shape, and location in CA1 of the hippocampus. Single neurons were cut out by laser capture under 20X objective. Neurons were dropped into inverted microcentrifuge caps containing proprietary buffer from ThermoFisher. Three single neurons were cut from AD brain and each one loaded into one of three microcentrifuge caps. Two single neurons from control brain were obtained similarly. The laser capture process was repeated to form caps with 10 neurons in triplicate from AD and control brain as well as one sample of 100 neurons from AD and another from control brain.

### Ion AmpliSeq™ Transcriptome Human Gene Expression

Libraries for RNA sequencing were prepared using AmpliSeq Library kit as previously described [17]. Briefly, total RNA was reverse transcribed into cDNA. Targeted genes were amplified with the Ion AmpliSeq human transcriptome panel, which contains a pool of oligonucleotide primer pairs, each pair designed to amplify a specified genome region. The panel designed for this study targeted 18,574 coding genes and 2,228 non-coding genes based on UCSC hg19 annotation. The panel contained 20,802 amplicons (41,604 primers), approximately 150 bases long, in a single pool. Only one amplicon was designed for each gene which means the design is on the common exon shared by multiple isoforms. Following PCR amplification, the amplicons were partially digested with FuPa enzyme and ligated with sample specific barcoded adapters. After removing remaining primers and other residual reaction components, purified amplicon libraries from each sample were combined and were sequenced on Ion Proton^TM^ sequencer (Thermo-Fisher), with HiQ sequencing chemistry.

**Table 1:** Summary of AD neuron transcriptome.

Samples	Mapped reads	On target	Targets detected	Genes Identified
Single neuron_1	5,416,542	90.65%	61.51%	15,094
Single neuron_2	5,871,995	81.56%	64.82%	15,586
Single neuron_3	5,748,512	67.48%	64.16%	15,582
10 neurons_1	6,153,109	83.52%	66.98%	16,375
10 neurons_2	5,793,326	83.08%	65.61%	16,075
10 neurons_3	5,596,528	84.35%	65.69%	16,662
100 neurons	4,239,544	89.57%	64.90%	16,764

### Data analysis

AmpliSeq sequencing data were analyzed using the Ion Torrent Mapping Alignment Program (TMAP) and were normalized using reads per million (RPM). Principal component analysis was performed using R program. Genes with >2-fold change of mean RPM values between AD and control neurons were considered as significantly changed and their biological functions were annotated by David Bioinformatics Resources [21]. The significance of the differential expression between AD and control brain was evaluated by Student’s t test followed by multiple testing correction with the false discovery rate (FDR) method. FDR greater than 0.05 was considered as statistically significant.

**Figure 1.**
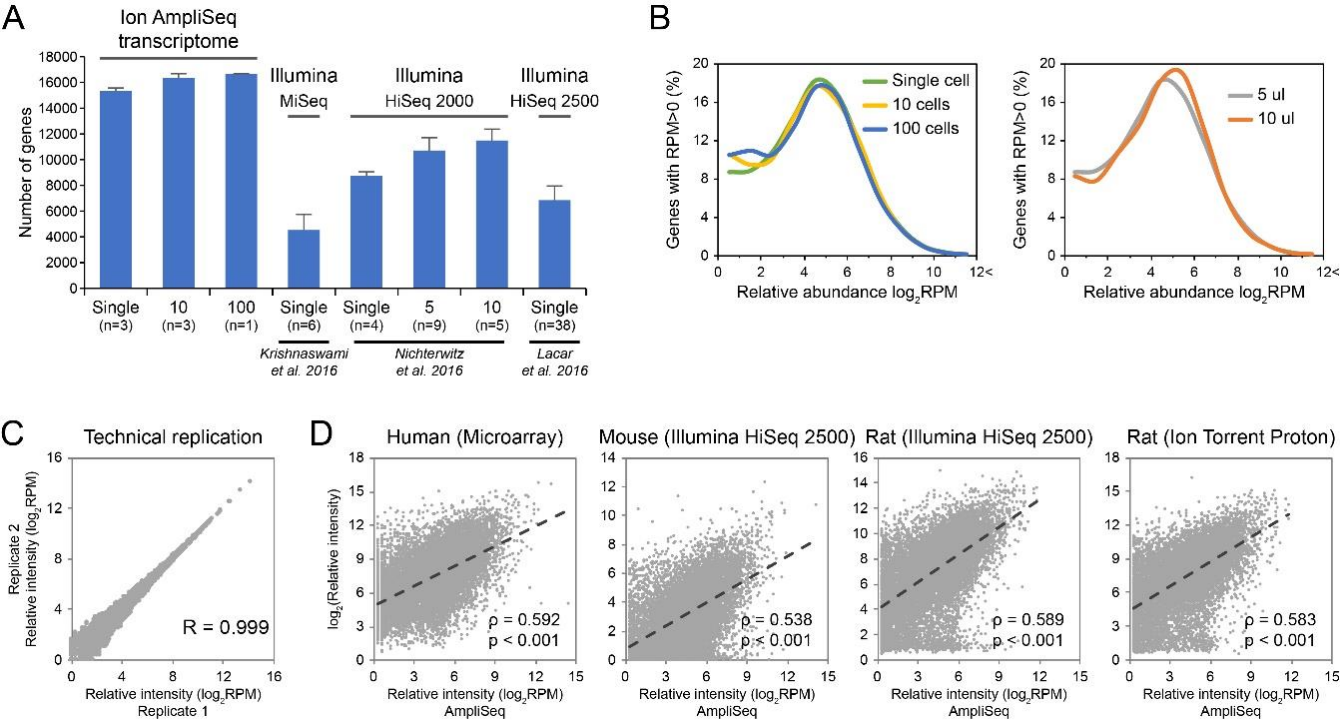
Evaluation of AmpliSeq transcriptome sequencing results. (A) Comparison of gene identifications between Ion AmpliSeq technology and other RNA sequencing platform. (B) The distribution of gene abundance in single neuron and pooled 10 and 100 neurons (left) as well as in single neurons with different loading amount (right). (C) Pearson correlation of gene expression between technical replicates. (D) Comparison of gene expression quantified by AmpliSeq with the hippocampus neuron transcriptome measured in other species using different technologies.

**Figure 2.**
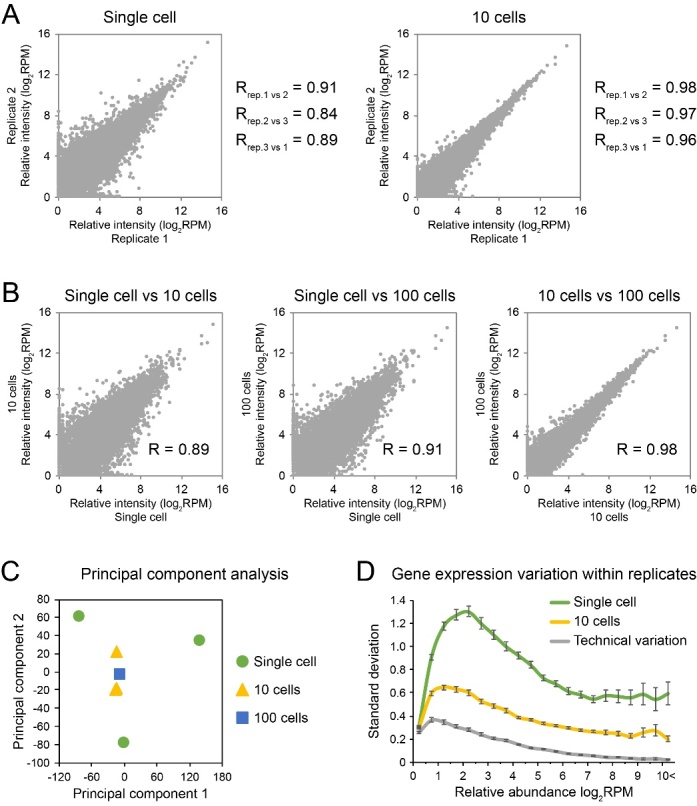
Gene expression diversity in different neuron sets. Pearson correlation of gene expression between replicate experiments (A) and across different neuron sets (B). (C) Principal component analysis of gene expression profiles in different neuron sets. (D) Standard deviation of gene expression within replicates binned according to gene expression levels (mean±95% CI).

## RESULTS

### Evaluation of AmpliSeq neuron transcriptome in AD

With the AmpliSeq technology, an average of 5.7 million reads were obtained for each single neuron. 67~91% of these reads (3.9~4.9 million) were on target and led to the identification of >15,000 genes in every single neuron, which was comparable to the average 16,371 genes identified in the 10-neuron pool and 16,764 genes in the 100-neuron pool (Table 1 and Table 2). As compared with other RNA sequencing studies, which used different platform (Illumina MiSeq and HiSeq) to profile single neuron or neuron pools at a similar scale to our study [22-24], Ion AmpliSeq technology largely increased the gene identification coverage, even at single cell resolution (Fig. 1A and Table 2). Moreover, similar abundance distribution was found for the genes identified in single neurons as well as in pooled 10 or 100 neurons (Fig. 1B). This remained robust even with different sample loading (5 μl vs. 10 μl - Fig. 1B). All these results may support the reliability of the single cell data and also suggest sufficient sensitivity and feasibility of AmpliSeq for single cell sequencing.

In addition, a high gene expression correlation was observed between technical replicates, indicating the reproducibility of AmpliSeq technology (0.999) (Fig. 1C). Significantly correlated gene expression can also be identified when comparing our data with the hippocampus CA1 neuron transcriptome measured in other species (human, mouse, rat) using different technologies (Microarray, Illumina RNA sequencing, Ion Torrent Proton) [25-27], which further confirmed the quantification accuracy and robustness of the AmpliSeq method (Spearman rank correlation: 0.592, 0.538, 0.589, 0.583; p<0.001) (Fig. 1D).

**Table 2:** Comparison with other single neuron sequencing studies.

Platform	Neuron origin	Neuron sets	Replicates	Identified genes			
				Minimum	Maximum	Average	SD
ThermoFisher Ion AmpliSeq transcriptome	Human hippocampus CA1	Single cell	3	15,094	15,586	15,421	283
		10 cells	3	16,075	16,662	16,371	294
		100 cells	1	N/A	N/A	16,764	N/A

Illumina MiSeq *Krishnaswami et al. 2016*	Human brain	Single cell	6	3,385	6,267	4,567	1,209

Illumina HiSeq 2000 *Nichterwitz et al. 2016*	Human motor neuron	Single cell	4	8,455	9,208	8,795	310
		5 cells	9	8,687	12,237	10,772	1,040
		10 cells	5	10,605	12,500	11,482	921

Illumina HiSeq 2500 *Lacar et al. 2016*	Mouse dentate granule cell	Single cell	38	4,553	8,769	6,843	1,135

### More diverse gene expression profile in single neurons than in pooled neurons

**Figure 3.**
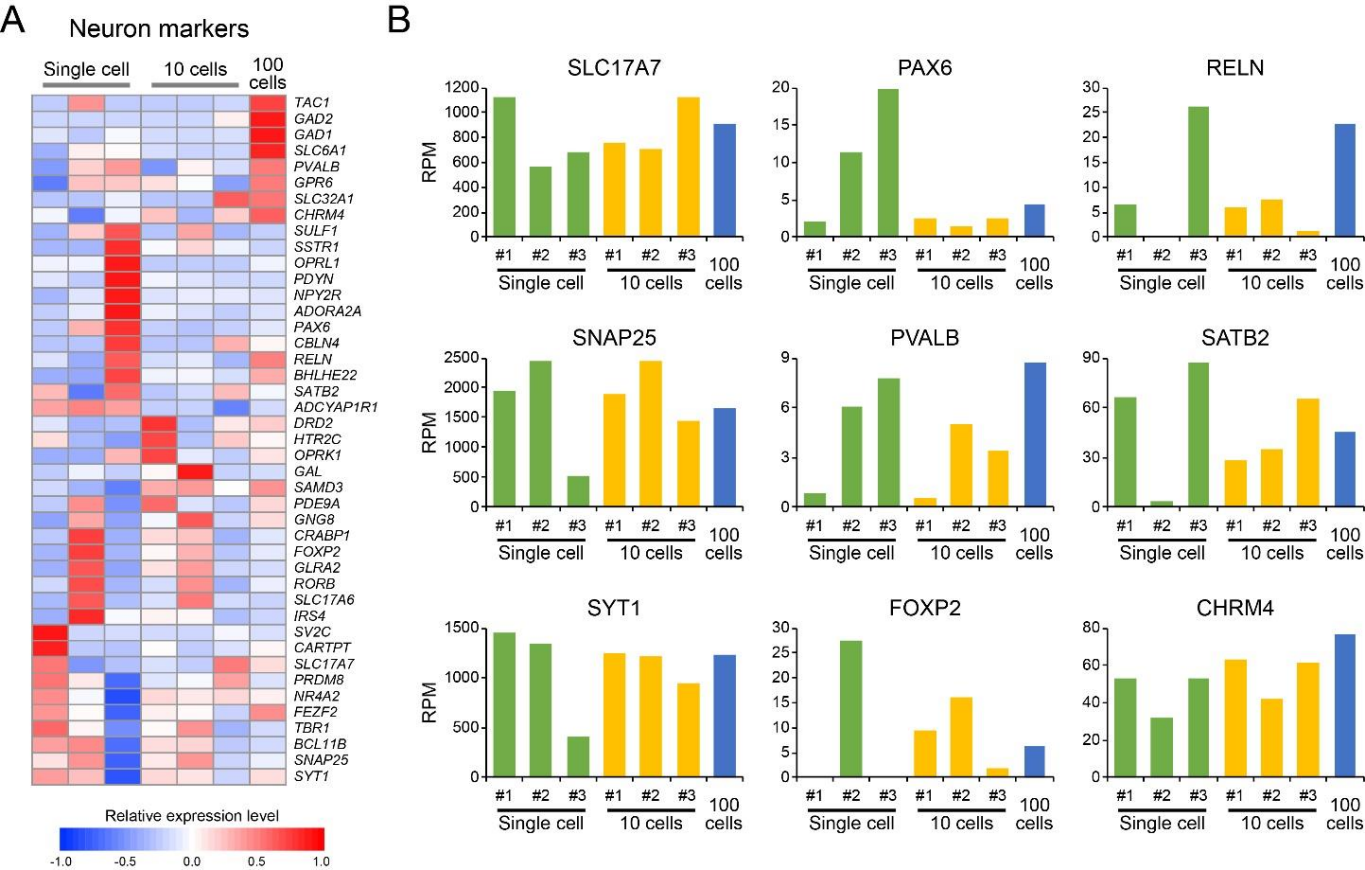
The expression diversity of neuronal markers in different neuron sets. (A) The relative expression of neuron markers across the different neuron sets. (B) Examples of neuron marker expression in single neurons and pooled 10 and 100 neurons.

We then compared the gene expression profiles across different neuron sets. As suggested by the relatively low correlation between replicates (0.91, 0.84, 0.89), single neurons demonstrated diverse gene expression patterns (Fig. 2A). This diversity could be reduced by pooling neurons into sets of 10 or 100, which could be evidenced by the increased correlation of gene expression between replicates of pooled 10 neurons (0.96, 0.97, 0.98) (Fig. 2A) as well as between 10 neurons vs. 100 neurons (0.98) (Fig. 2B). The reduced diversity from pooled neurons is further indicated by principal component analysis in which 10-cell and 100-cell pools were concentrated together, while larger distance was found between single neurons (Fig. 2C). Moreover, single neurons demonstrated much higher gene expression variation than the neuron pools and the technical replicates throughout the whole genome range (Fig. 2D). And many neuronal markers, which were used to discriminate different neuron subtypes [28, 29], were more differentially expressed in single neurons than the neuron pools (Fig. 3). We therefore concluded that the neuronal composition is highly heterogeneous in the hippocampus of AD patient and Ion AmpliSeq technology is capable of capturing these diversities and can be used to investigate AD-related alterations at a single cell level.

### Neuron transcriptome in AD versus Control

**Figure 4.**
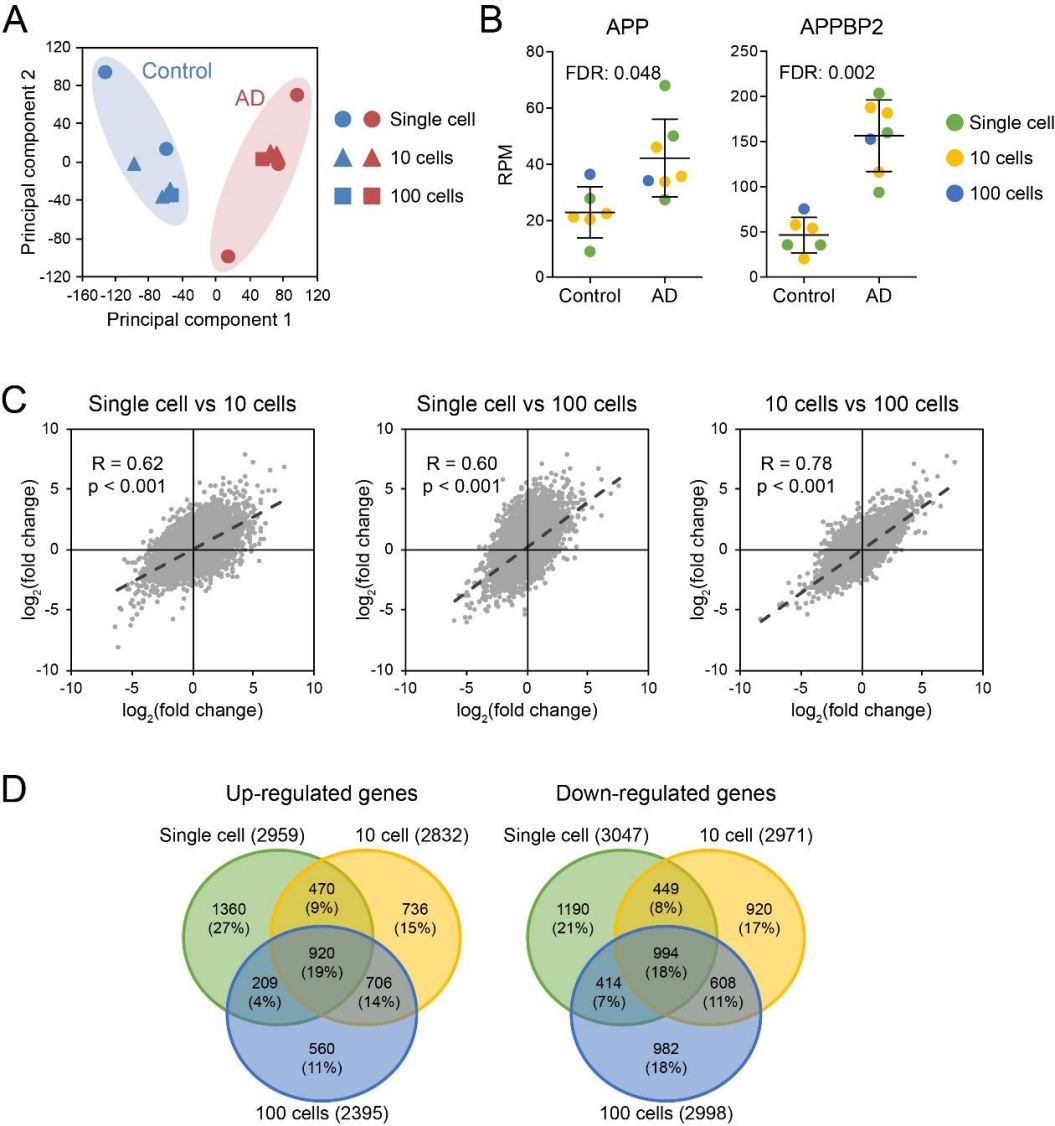
AmpliSeq neuron transcriptome in AD versus control. (A) Principal component analysis of gene expression between AD and control neurons. (B) The expression levels of well-characterized AD genes in AD and control neurons. (C) Correlation of gene expression changes across different neuron sets. (D) The Venn diagram of the genes with expression change in AD brain across different neuron sets.

Although single neurons exhibited distinct gene expression patterns, more dramatic differences were observed between samples from AD versus control brains. As shown in principal component analysis (Fig. 4A), neurons from AD patients and controls can be easily separated. Gene expression changes in AD exhibit a significant positive correlation between single neurons and neuron pools of 10 or 100 neurons (Fig. 4C). Moreover, using a cutoff of > 2-fold change, around 45% of the changes in AD were shared across different neuron sets (Fig. 4D). The expression of APP (amyloid precursor protein) and APPBP2 (APP binding protein 2), which are well characterized genes related to AD, were both significantly up-regulated in single, as well as pooled, AD neurons compared to neurons from control brain (Fig. 4B). All these results suggested that the major changes related to AD are conserved in single hippocampal neurons.

Of note and as expected, single neuron data defined a distinct AD space versus a distinct control space in the principal component analysis, which reflects the detectable population effects of AD in spite of diverse expression patterns of single neurons (Fig. 4A). When the diversity of single neurons is eliminated by pooling, the expression correlation between data from 10 vs 100 neurons (0.78) is higher that correlations for single neurons (0.62 and 0.60) (Fig. 4C).

### Functional alterations in single AD neuron and pooled neuron sets

**Figure 5.**
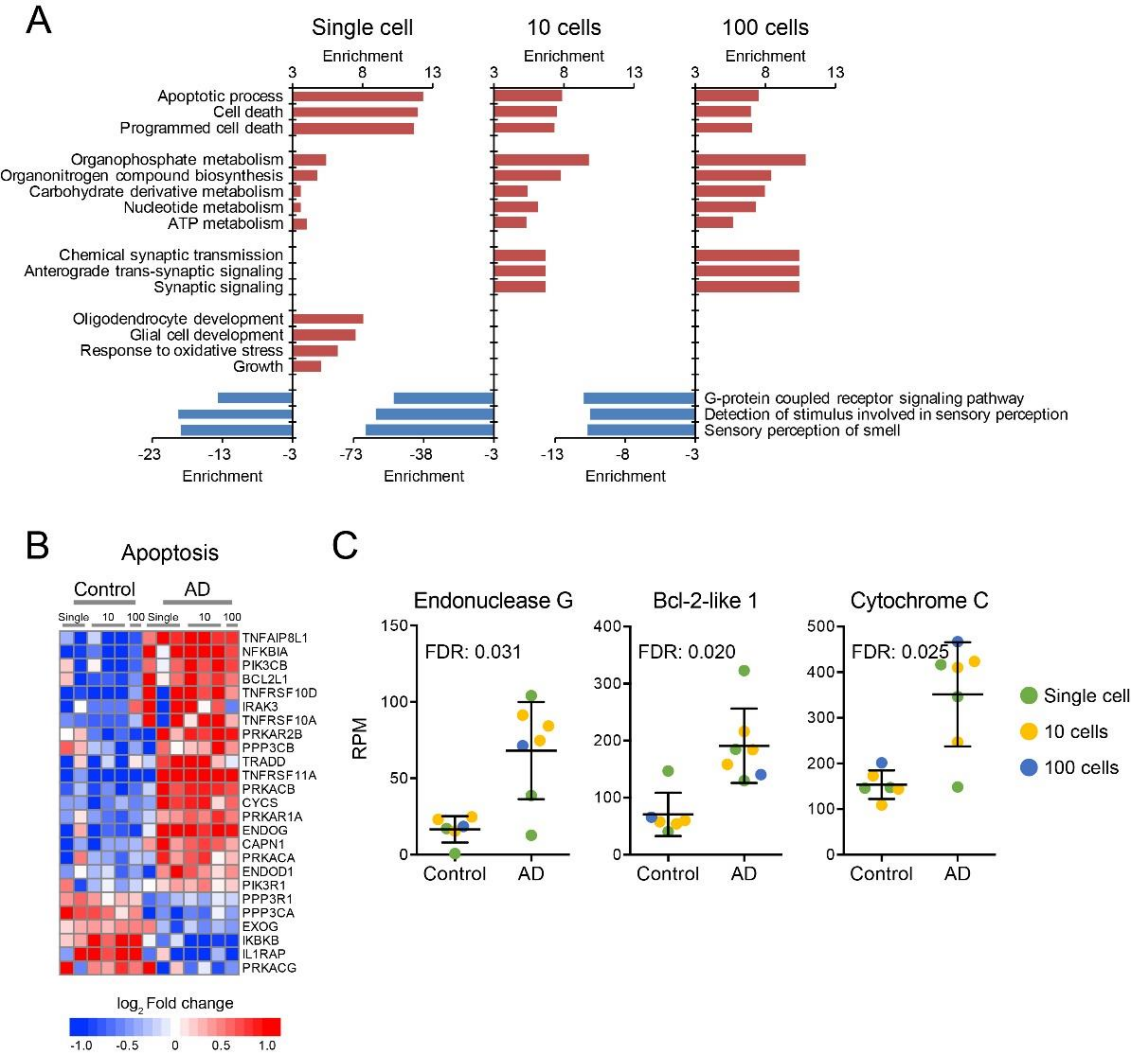
AD-related functional alterations in different neuron sets. (A) Biological functions enriched with the genes with >2-fold expression changes in AD brain. (B) The heatmap of genes involved in apoptosis. (C) The expression levels of well-characterized pro-apoptotic genes in AD and control neurons.

We then evaluated the functional alterations in AD with significantly changed genes in single neurons and neuron pools (> 2-fold change). As shown in Figure 5A, many of these changes are known to be affected in AD. Thus, we see involvement of cell death, metabolic processes, mitochondria, synapses, etc. Apoptosis was consistently upregulated in single AD neurons as well as in pooled 10 and 100 neurons with elevated expression for most of the genes involved in apoptosis (Fig. 5B). Notably, pro-apoptotic genes, endonuclease G, Bcl2 like 1 and cytochrome C were all significantly up-regulated, suggesting enhanced neuron death in AD brain (Fig. 5C). In contrast, sensory response and G-protein receptor signaling was extensively inhibited in single and pooled AD neurons (Fig. 5A). Moreover, the metabolic processes and synaptic signaling were increased in pooled AD neurons but were less affected in single neurons, suggesting that the small number of single neurons sampled were closer to dying with reduced metabolism and synaptic activity (Fig. 5A). On the other hand, the upregulation of oligodendrocyte development, glial cell development and oxidative stress response pathways was only found in single neurons, but not in pooled neurons (Fig. 5A). As a whole, these data suggested that single cell sequencing with the Ion AmpliSeq system can not only produce consistent data with pooled cell population, but also uncover single neuron responses that are hidden in pooled neuron data sets.

### Olfaction was extensively inhibited in AD hippocampus

**Figure 6.**
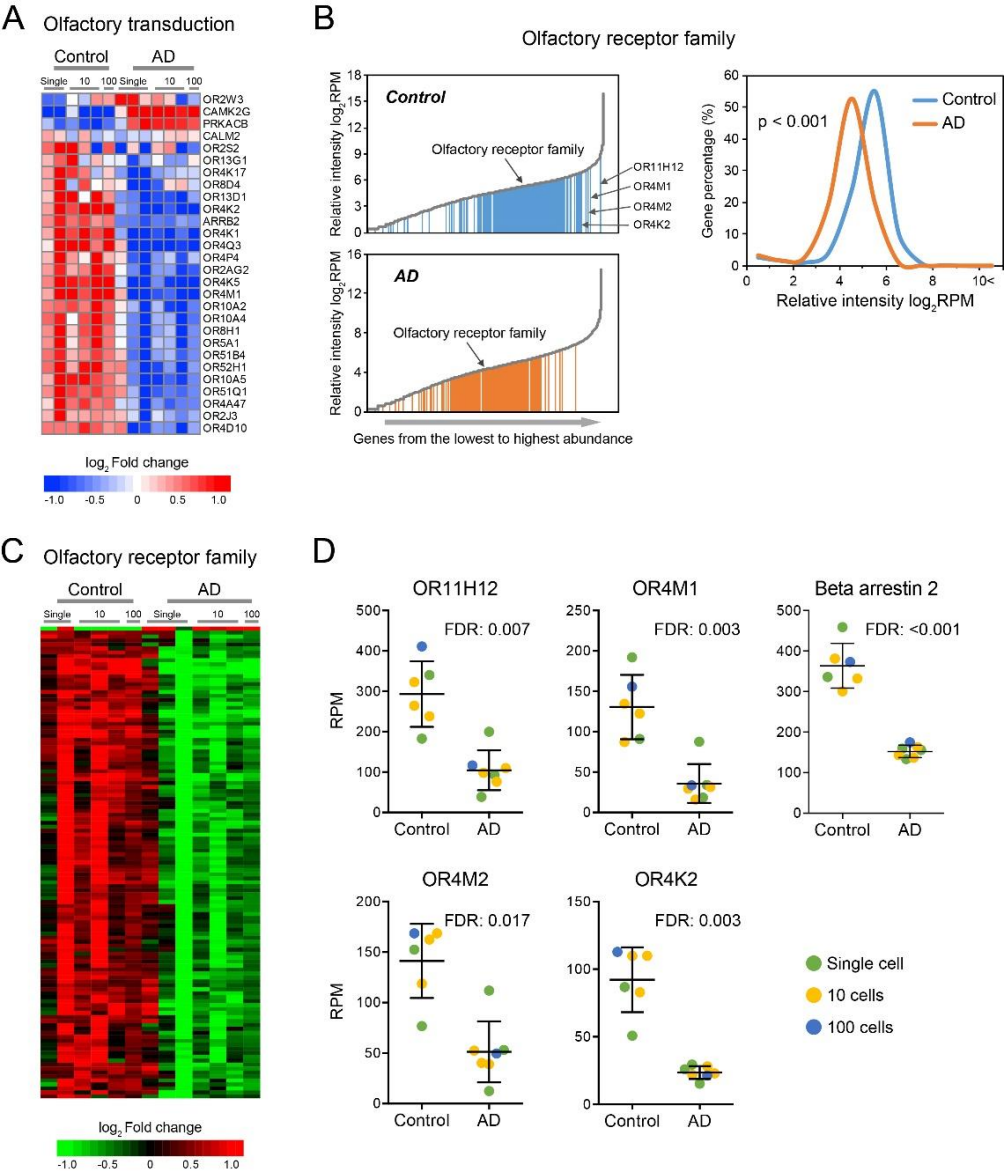
Olfactory transduction was significantly inhibited in AD neuron. (A) The heatmap of genes involved in olfactory signal transduction. (B) The expression distribution of olfactory receptor family in the genome of control and AD neuron. (C) The heatmap of significantly changed olfactory receptors. (D) The expression levels of the olfactory receptors with the highest abundance as well as β-arrestin 2 in AD and control neurons.

According to the functional analyses, olfactory system is largely reduced in the hippocampus of AD brain. As shown in Figure 6A, the inhibited olfactory response was mainly driven by the down-regulation of olfactory receptors. Impaired olfactory responsiveness is an early symptom of AD. Although these neurons are not directly involved in olfaction, the hippocampus does aid in olfactory memory. In our study, a total of 367 olfactory receptors were identified in hippocampus, which were expressed at moderate level in the genome of control neurons, implying the functional importance of hippocampus in olfaction (Fig. 6B). However, in AD hippocampus, the expression of the olfactory receptor family was generally suppressed (Fig. 6B). 119 family members (32.43%) demonstrated significantly decreased expression while only 1 member (0.27%) was increased (Fig. 6C). Of note, olfactory receptors with the highest abundance (OR11H12, OR4M1, OR4M2, OR4K2) were all significantly down-regulated in AD (Fig. 6B, 6D). Moreover, β-arrestin 2 (ARRB2), a crucial signal transductor of olfactory signaling, was also significantly reduced (Fig. 6A, 6D), suggesting the overall deactivation of olfactory signal transduction in AD hippocampus.

### Neurotransmitter receptors were reduced in AD hippocampus

**Figure 7.**
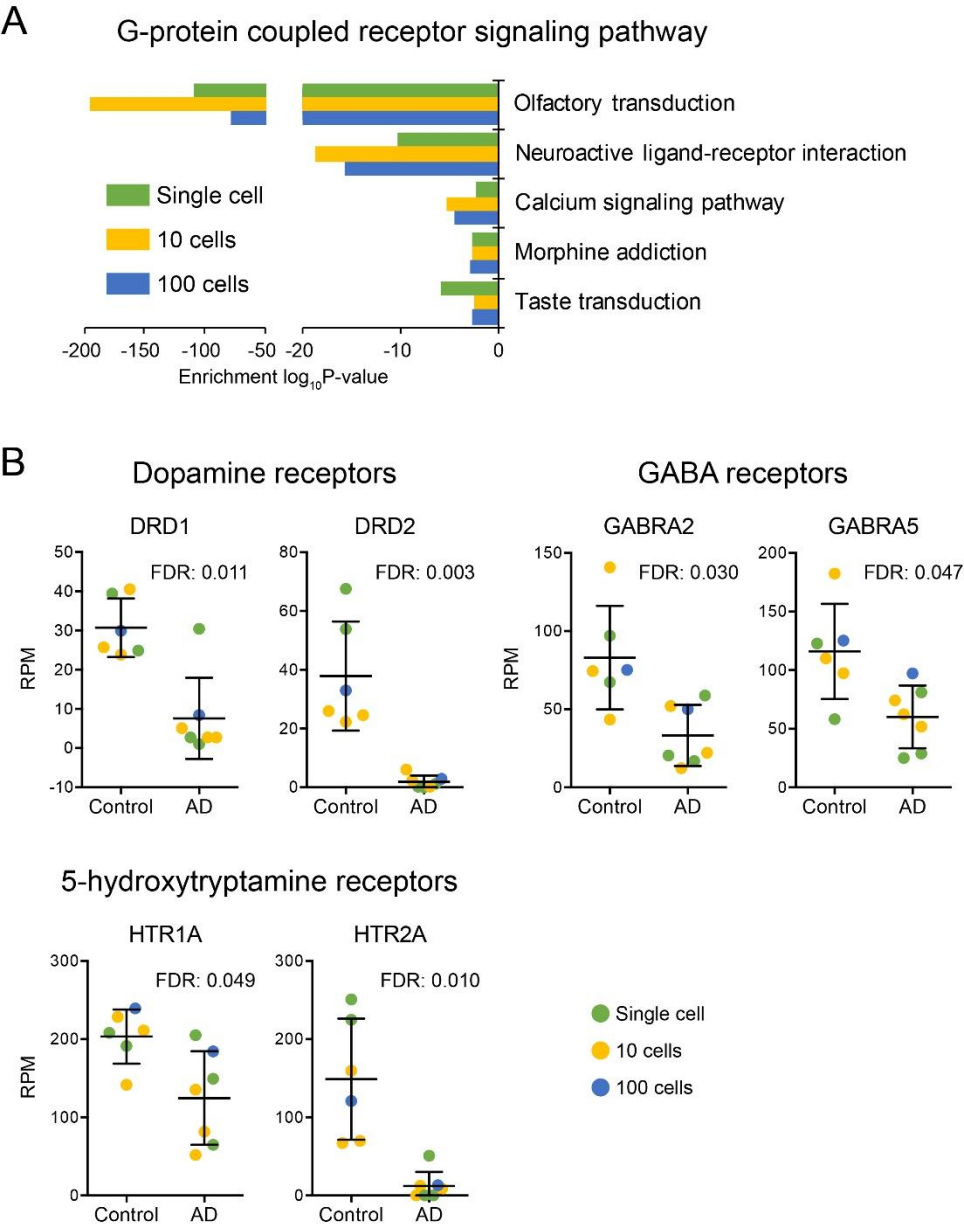
Neurotransmitter receptors were reduced in AD neurons. (A) The functional classification of the genes involved in G-protein coupled receptor signaling pathway. (B) The expression levels of the receptors of dopamine, GABA and 5-hydroxytryptamine neurotransmission system.

In addition to the olfactory transduction, other G-protein coupled receptor signaling pathways were also inhibited in AD (Fig. 7A), including neural transmission mediated by neuroactive ligand-receptor interaction. Multiple previous studies have shown inhibited neural transmission system in AD. Consistently, in our study, we found that the receptors of important neural transmission systems, including dopamine receptors (DRD1, DRD2), GABA receptors (GABRA2, GABRA5) and 5-hydroxytryamine receptors (HTR1A, HTR2A), were all significantly down regulated in AD neurons (Fig. 7B). It is notable that no decrements were detected for the corresponding transmitters (ligands) of these systems. Decrements in receptors for important transmission systems in the absence of corresponding decrements of transmitter synthesis suggests that AD therapeutic interventions targeted at increasing transmitter availability must be ineffective in the face of reduction in the ability to receive these transmitters.

## DISCUSSION

The major purpose of the work described here was to determine whether the ThermoFisher Ion AmpliSeq system could be used to obtain valid data from single neurons or small sets of neurons obtained by laser capture microdissection from postmortem human brain. We profiled the gene expression in single CA1 neurons as well as sets of 10 or 100 pooled neurons that were laser captured from AD and control hippocampus. We found that the Ion AmpliSeq approach is robust and sensitive in measuring transcriptome at a single cell resolution. With the targeted nature of the AmpliSeq transcriptome, more than 15,000 genes were identified with very small RNA input from each single neuron, which covers a more comprehensive genome compared to the 4,000~9,000 identifications per cell by other single neuron sequencing technologies [22-24]. Since laser capture microdissection limited the section thickness to 15 µm, smaller than that of a neural cell, only a portion of the RNA was captured from each single neuron, which further confirmed increased sensitivity of the AmpliSeq method. Moreover, the data obtained from AmpliSeq system were both internally consistent among single neurons and pooled neuron sets, as well as consistent with changes in gene expression known to typify AD neurons from affected brain regions. Known changes in AD are associated with alterations in cell death [30-32], metabolic activity [33-36], synaptic structure and function [37-39], transmitter receptors [40], and olfaction [41, 42] - all of these responses were detected in our data from single neurons as well as pooled sets of 10 or 100 neurons.

Additionally, we found consistent reduction in AD hippocampal CA1 neurons of genes related to olfactory system, particularly the reduction of the olfactory receptor family. Although decrements in olfactory function in AD are well known [41, 42], global decrements in expression of genes related to olfaction in CA1 of hippocampus have not received much emphasis. However, there is ample evidence to associate the hippocampus with decreased olfactory function in AD. Imaging studies showed reduced hippocampal volume to be associated with decreased ability to identify odors [41] and morphological evidence documented projections from the olfactory bulb to the hippocampus [43]. Additionally, electro-physiological data have demonstrated potentials evoked in the hippocampus by stimulation of the olfactory bulb [44]. In addition to olfactory receptors, we also provided evidence that the receptors, but not the transmitters, of other neural transmission system (dopamine, GABA and 5-hydroxytryamine receptors) were also coordinately downregulated in AD hippocampal neurons. These results might have therapeutic implications; increasing the sensitivity of receptors may be more clinically effective than simply increasing the level of neurotransmitters per se.

Importantly, there are classes of transcripts that disappear as data moves from single neurons to pooled sets of neurons, and the converse - class(es) that appear as data moves from single neurons to sets of neurons. In the case of the former (glial cell development and response to oxidative stress) we assume that these are processes that happen to be present at relatively low levels in the small number of single neurons profiled, but then become swamped when more neurons are added to the mix. In the case of processes that are absent in the single neurons sampled, but increasingly present as more neurons are added to the mix (e.g. metabolic processes and synaptic transcripts) we consider these data in the context of the data on apoptotic processes and cell death in the single neurons we have selected. This suggests that the small number of single neurons selected were on the way toward cell death, and therefore, reducing expression of metabolism and synapse-related transcripts. As more neurons are added to the analysis, cell death signals decrease, metabolic processes increase, suggesting healthier neurons and synapse transcripts increase. The differences between single cell data and data from sets of 10 or 100 neurons emphasize the heterogeneity of single neuronal responses and the need for more data at the single cell level in order to truly capture the complex pathophysiology that is triggered in AD. Nevertheless, data such as those in the PCA and heat map analyses also suggest that for the present data, this heterogeneity/diversity exists within broader frameworks that may allow distinction of pertinent classes such as disease versus healthy states.

There are a few caveats. As an exploratory study, the sample size used here was limited. Although our data show internal consistency as well as the consistency with the literature, the details of the results describing differential gene expression between AD and control brain need to be verified in more neurons from more patients and cannot be considered as stand-alone descriptions of the AD brain. Using laser capture microdissection may lose information about the spatial localization of the cells selected. This loss may be partially mitigated by bar-coding cells selected for laser capture as well as by capturing more neurons across larger anatomic locales to get a more comprehensive view of AD-related neural alterations. Ultimately, further exploratory studies such as this should provide the basis for calculating power in order to pursue larger formal studies to compare single cell profiles and variations in AD versus normal aged brains.

### Conclusion

This proof-of-concept study suggests that the Ion AmpliSeq methodology presents a useful alternative to other methods of obtaining expression data from single neurons or collections of neurons.
